# Effect of lullaby on volume, fat, total protein and albumin concentration of breast milk in premature infants’ mothers admitted to NICU: a randomized controlled trial

**DOI:** 10.1186/s13006-022-00511-7

**Published:** 2022-09-29

**Authors:** Somayeh SefidHaji, Parvin Aziznejadroshan, Mohsen Haghshenas Mojaveri, Hossein-Ali Nikbakht, Durdi Qujeq, Seyedeh Roghieh Jafarian Amiri

**Affiliations:** 1 Student Research Committee, Babol University of Medical Sciences, Babol, Islamic Republic of Iran; 2 Non-Communicable Pediatric Disease Research Center, Health Research Institute, School of Nursing and Midwifery, Babol University of Medical Sciences, Babol, Islamic Republic of Iran; 3 Non-Communicable Pediatric Disease Research Center, Health Research Institute, School of Medicine, Babol University of Medical Sciences, Babol, Islamic Republic of Iran; 4 Social Determinants of Health Research Center, Health Research Institute, Department of Biostatistics & Epidemiology, School of Public Health, Babol University of Medical Sciences, Babol, Iran; 5 Department of Biochemistry, School of Medicine, Babol University of Medical Sciences, Babol, Iran; 6 Department of Nursing, School of Nursing and Midwifery, Babol University of Medical Sciences, Babol, Iran

**Keywords:** Breast feeding, Infant, Premature, Music, Intensive care units, neonatal

## Abstract

**Background:**

Listening to music can reduce or manage stress, fatigue, and accompanying symptoms in mothers. Music increases oxytocin secretion which affects breast milk. This study aimed to examine the effect of lullaby on volume, fat, total protein and albumin concentration of breast milk in mothers of premature infants admitted to the NICU.

**Methods:**

This clinical trial was performed on 100 primiparous mothers whose premature infants were hospitalized in the NICU of Ayatollah Rouhani Hospital from January 2020 to December 2020. Using block randomization method, the participants were divided into three groups: control (A), playing lullaby for mother (B) and playing lullaby for a mother while holding a photo of her own baby (C). The mothers of the intervention groups listened to lullabies through headphones for 30 minutes every morning for 6 days. On the first and the sixth day of birth, the volume of breast milk (ml) and two milliliters of breast milk samples of all three groups were measured and compared in terms of fat, albumin concentration and total protein (mg/DL). ANOVA, Paired T-Test and ANCOVA model (the included variables were: basic value of dependent variable, group type, Maternal age, Birth weight, Gestational age and Maternal weight) was used for analytical statistics.

**Results:**

The difference between the mean compositions of breast milk before and after the intervention in three groups of A, B and C: in terms of the breast milk volume were 66.33 ± 4.80, 71.30 ± 4.18 and 75.91 ± 6.80 ml; in terms of triglyceride level was 177.84 ± 50.57, 210.72 ± 34.55 and 224.17 ± 12.97 mg/DL, cholesterol level was 14.57 ± 3.70, 21.96 ± 3.82 and 26.26 ± 5.16 mg/DL, albumin concentration was 0.90 ± 0.30, 1.22 ± 0.19 and 1.46 ± 0.28 mg/DL and total protein level was 1.61 ± 0.61, 2.20 ± 0.57 and 2.72 ± 0.30 mg/DL. Finally, the results of ANCOVA analysis for the effects of the intervention, taking into account the baseline values, showed that the intervention was effective and had the greatest effect on cholesterol levels.

**Conclusion:**

In this small trial, there was a statistically significant association between trial arm and biochemical composition of breastmilk though further studies are needed to see if these changes result in meaningful clinical outcomes to the infant.

**Trial registration:**

IRCT, IRCT20191114045439N1. Registered 14 January 2020- prospective, https://en.irct.ir/trial/43671

**Supplementary Information:**

The online version contains supplementary material available at 10.1186/s13006-022-00511-7.

## Background

According to the World Health Organization (WHO), preterm birth is a live birth that occurs before 37 completed weeks of pregnancy [1]. The trends of preterm birth rates found that the global preterm birth rate rose form 9.8% in 2000 to 10.6% in 2014 [2]. Exclusive breast feeding (EBF) is a component of optimal breastfeeding practices effective in preventing child morbidity and mortality [3]. Despite the benefits, the proportion of exclusively breastfed children remains low in many low- and middle-income countries (LMICs), where most child deaths attributed to suboptimal breastfeeding occur [4]. At 48 hours, 43.3% women and 37.4% primiparous women were not breastfeeding [5]. About 38% of children worldwide who are under 6 months of age are exclusively breastfed from birth; this percentage has not increased notably in the past two decades [6]. The highest EBF rates are reported in eastern and southern regions in Africa (51%) [6]. In spite of the well-recognized importance of EBF, the practice is not widespread in the developing world and an increase on the global level is still very modest with much room for improvement [7]. The overall prevalence of EBF in Iran was 53% [8]. In Iran health policy- and decision-makers should try to take interventions that encourage mothers to use their milk to breastfeed the infants [8]. The American Academy of Pediatrics recommends human milk as the “gold standard” for infant feeding and nutrition, especially for premature infants [9]. The WHO has set a target to globally increase EBF rates during the first 6 months postpartum to 50% by 2025 [10]. Breast milk provides both long-term and short-term advantages for infants. In the short term, it protects against intestinal malnutrition, nosocomial infections and necrotizing enterocolitis, reduces the incidence of late sepsis, chronic lung disease, early retinopathy, and reduces the length of hospitalization and the rate of hospital readmission [11, 12]. In the long term, breast milk protects the child against diabetes, lymphoma, leukemia, and Hodgkin’s disease, also reduces obesity, high cholesterol, asthma, and improves cognitive development [13]. These benefits are related to the natural immunity mature breastmilk confers [11]. Milk from women who deliver prematurely differs from that of women who deliver at term [14]. Preterm milk was higher in true protein than term milk [15]. After postnatal day 3, most of the differences in true protein between preterm and term milk were within 0.2 g/DL, and the week 10–12 estimates suggested that term milk may be the same as preterm milk by that age [15]. Colostrum was higher than mature milk for protein, and lower than mature milk for energy, fat and lactose for both preterm and term milk. Breast milk composition was relatively stable between 2 and 12 weeks. With milk maturation, there was a narrowing of the protein variance [15]. Most breast milk fatty acids are in the form of triglycerides [9]. Mothers of premature infants are at risk of not producing adequate milk [16]. Maternal and neonatal factors have the greatest effect on EBF [17]. There are various reasons for low milk volume in mothers of premature infants. One of the common factors is the separation of the infant from the mother, especially in premature infants. When the infant is separated from the mother, it cannot stimulate the mother’s breast to produce milk, and less breast stimulation causes less milk production [18]. Another factor is the mother’s mental health. Stress and fatigue are considered to inhibit lactation, and mothers of premature infants are known to be three times more likely to experience clinically significant psychological distress than those of the normative population [19].

Maternal stress and fatigue reduce the function of oxytocin and reduce the milk ejection reflex, resulting in reduced milk production and low quality [19]. Oxytocin makes the myoepithelial cells around the alveoli contract. This makes the milk, which has collected in the alveoli, flow along and fill the ducts and the baby receives this milk from the nipple [20]. Oxytocin has a positive correlation with milk volume [21]; thus, interventions that increase oxytocin secretion could lead to higher milk volume [16]. Experts encourage premature infants’ mothers in a variety of ways, including education, emotional support, positive feedback on the baby’s growth and condition, training to use a breast pump, skin-to-skin care (kangaroo care), and relaxation techniques [22, 23]. Despite these interventions, human milk insufciency is a signifcant barrier to implementing breastfeeding, and it is identifed as a prevalent concern in 60–90% of mothers in low-and-middle-income countries. Breastmilk insufciency can lead to hypoglycemia, hypernatremia, nutritional defciencies, and failure to thrive in newborns and infants [24].

Music therapy offers a long history of clinical practice and research [16]. Listening to music can reduce or manage stress, fatigue, and accompanying symptoms in mothers [16, 25]. A meta-analysis of 22 quantitative studies revealed that music therapy is an effective means of reducing stress [25]. Music therapy can reduce stress in mothers with a premature baby in a neonatal ICU, and this can relatively increase breast milk production, which affects the development of premature babies [26]. Music increases oxytocin secretion which affects breast milk [18]. Listening to music, relaxing, warming the breast, constantly pumping the breast increases the secretion and volume of breast milk [27]. Lullaby is an appropriate choice of hypnotic and relaxing music and traditionally contains a predictable and well-known tone with a steady rhythm length of eight times, sixty to eighty beats per minute, short melodic phrases followed by a long pause, slow pace and many repetitions [28].

Given the high prevalence of preterm births, their long-term hospitalization in NICU and the high cost of treatment, the researchers thought that perhaps routine breastfeeding training combined with lullaby effects may increase the production and quality of breast milk, reduce the duration of treatment, play an effective role in faster neonatal discharge and in addition to cost-effectiveness, can reduce the risk of unwanted complications in preterm infants of primiparous mothers. Previous studies have looked more at the effect of music on increasing breast milk volume and less attention has been paid to the quality of breast milk (fat, protein and albumin). Due to breastmilk’s importance for growth and development, hence, quality is important [26]. Keith’s study showed the effect of music on the volume, fat and calories of breast milk in mothers of premature infants admitted to the NICU. The findings of this study indicated that music increases the volume, fat and calories of breast milk in the intervention group [16]. However, the results of the Varişoğlu’s study showed that music had no effect on increasing breast milk volume and there was no statistically significant difference between the intervention and control groups [29]. The results of the Hinesley’s study showed that exposure to a lullaby intervention was not statistically associated with maternal/fetal attachment, mental health, and perceived stress [30]. Therefore, due to the contradictory results regarding the effect of music on breast milk, the present study aimed to determine the effect of lullaby on volume, fat, total protein and albumin concentration of breast milk in mothers of premature infants admitted to NICU.

## Methods

### Study design and setting

This 3-armed randomized controlled trial study was performed on 100 primiparous mothers whose premature infants were born during the 11 months from January 2020 to December 2020 in the NICU of academic center (Rouhani Teaching Hospital, Babol, Iran). NICU of Rouhani Hospital has level III intensive care affiliated with Babol University of Medical Sciences and high-risk pregnancy referral center.

### Participants

The mothers who referred to the hospital were mostly local residents of the area and all of them were Persian-speaking individuals. Primiparous mothers based on the time of admission of their premature infants in the NICU of Ayatollah Rouhani Hospital were selected. Interventions for mothers were performed on the third day of neonatal hospitalization.

From the beginning of the study to the required sample size, Eligible individuals with eligibility criteria were included in the study. The mothers of the neonates signed an informed consent for inclusion in the study prior to the start of and before randomization.

The mother’s willingness to breastfeed, possibility of mother’s presence for breastfeeding at least once a day in the hospital, cesarean section mothers, mothers of normal hearing were included. Mothers due to infants with G.A < 34 weeks, mother due to neonates with significant congenital anomalies, mother refuses to participate, mothers with history of drug abuse or oral galactagogues and mother-child dyads with known contraindications for breastfeeding were excluded.

The reason for exclude infants (< 34) sucking are not Coordinated with swallowing and breathing [31], infants are fed with gavage or syringe, while late premature infants (34-37) are feed via the breast. Therefore, if we consider infants < 34 weeks, their feeding methods will be different.

For all cesarean section mothers, spinal anesthesia was used with markin injection (bupivacaine). For each mother, 2.2 cc of spinal anesthesia was injected. The effect of this drug is about 2 hours. 30 units of oxytocin per liter of Ringer serum were injected during labor in the operating room (15-20 minutes). Also, after transferring the mother to the ward, 30 units of oxytocin in one liter of Ringer serum were injected into the mother for 8 hours. Opioids were not used for cesarean section mothers.

### Randomization and blinding

Primiparous mothers were randomly allocated to one of three study groups: control (A) with 33 participants, playing lullaby for mother (B) with 33 participants and playing lullaby for a mother while holding a photo of her own baby (C) with 34 participants, through a using a computer random number generator and with a strategy of permuted blocks with size of six. To maintain randomization during the study, the allocation concealment method was performed. For allocation concealment so that the next person to be assigned to which group would not be known. They were only consecutively revealed in the final expulsion phase by the study monitor assigned to the center. The first author was approached the participants.

Blinding for nurses who implemented the intervention was not possible given the nature of the intervention. All outcomes in this study were measurement by laboratory instruments. Due to the characteristics of the study intervention, blinding was applied only to a group of researchers who were laboratory evaluators and data analysts. Incomplete blinding, but the review authors judge that the outcome is not likely to be influenced by lack of blinding.

**Fig. 1:**
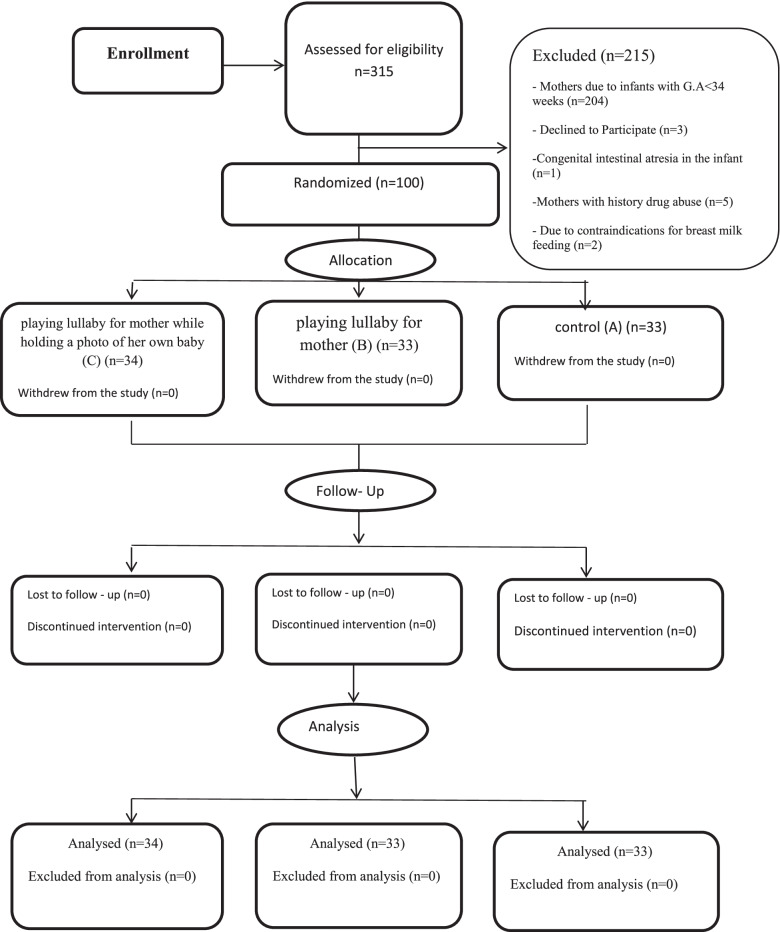
Participant flow diagram according to consolidated standards of reporting trials

This study followed the CONSORT guidelines for reporting randomized controlled trials (Fig.1).

### Sample size

The sample size was determined based on a study by Keith et al. [16] on the effect of lullaby interventions on increasing breast milk volume and fat. The mean difference (standard deviation) in the two groups was considered based on the same study of 46.2 (6.5) and 51.8 (12.2). The sample size was calculated based on the effect size. This effect size was obtained based on the mean difference before and after the intervention according to the same study.

The minimum sample size in the three groups (A, B, C) was calculated using G Power software version 3 [32], with a test power of 80% and an error of 0.05 and effect size of 0.53. A total of 90 people were required for the three groups (30 individuals for each group), and with 10% of the loss, 100 people were determined as the final sample size.

$$n=\frac{{\left({Z}_{1=a/2}+{Z}_{1=\beta}\right)}^2{(S)}^2}{{\left({\mu}_1-{\mu}_2\right)}^2}$$

$${Z}_{1-\frac{\alpha }{2}}=1.96\kern0.5em 0.84={Z}_{1-\beta}\kern0.5em 42.3={S}_1^2$$

$${\displaystyle \begin{array}{cc}148.8={S}_2^2& 46.2={\mu}_1\end{array}}$$

$${\displaystyle \begin{array}{cc}51.8={\mu}_2& \mathrm{SD}=\sqrt{\frac{\Big({S}_1^2+{S}_2^2}{2}}\end{array}}$$

Correlation between groups = 0.5.

### Data collection

The mothers in the control group first washed their hands and sat on a comfortable chair in the breastfeeding room after a routine breastfeeding training class conducted by a nursing expert working in the NICU. The operation of the electric milking pump in the ward was explained to the mothers of all three groups. Because the quality of breast milk changes during the day and the amount of milk fat in the morning is less, Keith et al. chose to do this process around noon because the stability of milk fat is higher at this time [16]. We also chose this time in our study, and besides, it was also in line with the time of mothers’ training. For all mothers in the control group, after the end of the routine breastfeeding training class, the milk was collected by an electric milking pump and measured in a graduated container based on milliliters under the supervision of researcher.

Mothers of groups B and C, in addition to participating in the routine breastfeeding training class, listened to traditional Iranian lullabies with a female voice through a headphone for 30 minutes [33] with an intensity of sixty-five to seventy-five decibels [34]. Mothers listened to lullabies for 15 minutes. At the end of the first 15 minutes, they started with electric milking pump and in the second 15 minutes, milking continued while the mother was listening to lullabies [28] in the presence of the researcher. In group C, in addition to the routine breastfeeding training class and playing lullabies, mothers look at the photo or their baby. The photo of the baby was taken by the researcher with a 16-megapixel camera at a distance of 30 cm without flashing, while the baby was sleeping. It was taken with the permission of the baby’s parents and doctor. The photos of the babies were pre-printed and given to the mothers of the babies in group C only during the intervention. The timeframe was 0-6 days (one session every day). None of the mothers in the three groups were trained separately.

### Outcomes assessment

The primary outcome was the effect of lullaby on the volume of breast milk in mothers of premature infants. For this purpose, Breast milk in all three groups was collected on the first and sixth day of the study around noon (11:30-12:00 AM) using a Mamivac electric pumping machine made in Germany and the volume of breast milk was measured by Medela graduated glass container (capacity 100 cc) made in Switzerland based on milliliters in all three groups. Milk volume was measured by the sixth researcher and recorded in a questionnaire.

The secondary outcome was to determine the effect of the lullaby on cholesterol, triglyceride, total protein and albumin concentration of breast milk in mothers of preterm infants. For this purpose, the researcher collected 2 ml of breast milk from all three groups on the first and the sixth day of the study and transferred to the Biochemistry Laboratory of the Babol University of Medical Sciences to compare the biochemical indicators of breast milk in terms of fat (cholesterol and triglyceride), albumin and total protein using a Spectrophotometer with accurate measurement of 1 mg/DL performing by a fixed laboratory expert according to the instructions on the standard test kit of Pars Azmoon (made in Iran) and based on mg/DL (Table 1).

**Table 1:** Characteristics of instruments

Instruments	Model	Country	Serial No	CE
Spectrophotometer	UNICO, 2100	England	S/N080530, A 0801064	Power source AC220v/50 Hz, Fuse 220v/315A Fast-Acting
Centrifuge	Clements 2000	Australia, Sydney	R2015	Cycle 50,Volts 240,Amps 3.5

Infant demographic information (sex, age, birth weight) was collected from the hospital record, maternal characteristics (age, weight, and economic status) by self-reporting.

### Statistical analysis

The statistics advisor performed the data analysis blindly using SPSS Version 20 and a *p*-value< 0.05 was considered significant. Descriptive information was shown as frequency, percentage, mean and standard deviation.

To evaluate the related statistical tests, first, the normality of the data was evaluated using the Kolmogorov-Smirnov test and parametric tests were used due to its normality. To evaluate the relationship of two grouped variable, the chi-square test and paired t-test were used to examine the mean difference between the groups before and after the intervention. ANOVA was also used to compare the mean differences of quantitative variables between the three groups. Finally, the ANCOVA model was used to evaluate the effects of intervention for breast milk compounds with adjusted for baseline values [35].

## Results

### Study subjects

The total number of mothers who gave birth during the 11 months from January 2020 to December 2020 was 1624, but the total number of mothers whose babies were admitted to the NICU was 315. Of the 315 mothers, 215 mothers were excluded (3 mothers declined to Participate, 2 due to contraindications for breast milk feeding in the mothers, 5 mothers with history drug abuse, 1 mother due to congenital intestinal atresia in the infant, 204 mothers due to infants with G.A < 34 weeks). A total of 100 mothers were included: (A) control group with 33 participants; (B) playing lullaby for mother with 33 participants and (C) playing lullaby for mother while holding a photo of her own baby with 34 participants (Fig. 1).

75% of mothers had moderate economic status. There was no significant difference between the three groups in terms of economic status, maternal age, birth weight, fetal age and maternal weight (Table 2).

**Table 2:** Comparison of demographic characteristics of the groups

Groups^*^	Variable	Maternal age (year)	Birth weight (g)	Gestational age (week)	Maternal weight (kg)	Economic Status		
						Low n(%)	Moderate n(%)	High n(%)
A	SD ± Mean	29.76 ± 4.80	1681.81 ± 462.44	34.84 ± 0.83	5.52 ± 60.57	4 (12.12)	21 (63.63)	8 (24.24)
B	SD ± Mean	28.94 ± 3.14	1739.69 ± 357.19	34.81 ± 0.98	62.24 ± 7.47	0 (0.00)	30 (90.90)	3 (9.09)
C	SD ± Mean	30.82 ± 3.56	1705.29 ± 332.62	35.05 ± 0.95	64.05 ± 10.36	2 (5.88)	24 (70.58)	8 (23.53)
F		1.96	0.18	0.67	1.56	**χ**^**2**^:0.22		
df		99	99	99	99	1		
*P*_value_		0.14	0.83	0.51	0.21	0.63		

Groups^*^: A. Control, B. playing lullaby for mother,C. playing lullaby for mother while holding a photo of her own baby

In all three groups, the volume, triglyceride, cholesterol, albumin and total protein of breast milk in mothers of premature infants had statistically significant differences before and after the intervention (*p* < 0.001). Part of this difference could be due to the natural process of increasing breast milk on the sixth day after birth compared to the first day. Therefore, the mean difference (MD) was used to show the difference between the intervention groups (Table 3).

**Table 3:** Mean difference, Mean and standard deviation of the volume, triglyceride, cholesterol, albumin and total protein of breast milk in mothers of preterm infants admitted

Groups^**^	Variable	Time of intervention	Volume ml	Triglyceride mg/DL	Cholesterol mg/DL	Albumin mg/DL	Total protein mg/DL
A	SD ± Mean	Before	6.75 ± 0.93	388.72 ± 70.54	93.69 ± 20.96	1.29 ± 0.40	3.2 ± 1.11
		After	73.09 ± 5.41	566.57 ± 79.49	108.27 ± 18.98	2.19 ± 0.47	4.81 ± 1.00
		MD^*^ (%95 CI)	66.33 (64.62, 68.03)	177.84 (159.91, 195.78)	14.57 (13.26, 15.89)	0.90 (0.79, 1.01)	1.61 (1.39, 1.83)
t, df			79.27, 32	20.19, 32	22.57, 32	17.00, 32	15.00, 32
*P* value			< 0.001	< 0.001	< 0.001	< 0.001	< 0.001
B	SD ± Mean	Before	6.66 ± 0.98	384 ± 78.02	93.12 ± 20.24	1.28 ± 0.46	3.18 ± 1.08
		After	77.97 ± 4.47	595.36 ± 78.23	115.09 ± 20.58	2.51 ± 0.50	5.38 ± 1.18
		MD (%95 CI)	71.30 (69.81, 72.78)	210.72 (198.47, 222.97)	21.96 (20.61, 23.32)	1.22 (1.15, 1.29)	2.20 (1.99, 2.40)
t, df			97.83, 32	35.03, 32	33.03, 32	36.61, 32	21.79, 32
*P* value			< 0.001	< 0.001	< 0.001	< 0.001	< 0.001
C	SD ± Mean	Before	6.67 ± 0.91	386.73 ± 79.61	92.91 ± 17.39	1.24 ± 0.39	3.07 ± 0.92
		After	82.58 ± 7.02	610.91 ± 78.63	119.17 ± 17.81	2.71 ± 0.49	5.79 ± 0.97
		MD (%95 CI)	75.91 (73.53, 78.28)	224.17 (219.64, 228.70)	26.26 (24.46, 28.06)	1.46 (1.36, 1.56)	2.72 (2.61, 2.82)
t, df			65.07, 33	100.75, 33	29.64, 33	29.51, 33	52.82, 33
*P* value			< 0.001	< 0.001	< 0.001	< 0.001	< 0.001

to NICU before and after intervention

* Mean difference (Confidence interval)

Groups^**^: A. Control, B. playing lullaby for mother, C. playing lullaby for mother while holding a photo of her own baby

The results showed that volume, triglyceride, cholesterol, albumin and total protein of breast milk in mothers of premature infants were higher in the group (C) than group (B) and group (B) than group (A). Based on the results of paired t-test, it was found that in all groups, the values of five compositions of breast milk increased after the interventions as compared before the intervention, which was observed in all compositions and all groups and the difference was statistically significant (Table 3).

Then, results found that this difference also exists in comparison of the groups (*P* < 0.001) (Table 4).

**Table 4:** Comparison of Mean difference between the volume, triglycerides, cholesterol, albumin and total protein in breast milk before and after the intervention

Variable	Group^*^	Mean difference (MD)	Confidence interval %95 CI	Standardized effect size	F, df *P* value (Total)
Volume (ml)	B-A	4.96	1.80, 8.13	1.10	26.36, 99 < 0.001
	C-A	9.57	6.43, 12.71	1.62	
	C-B	4.60	1.46, 7.74	0.81	
Triglycerides (mg/DL)	B-A	32.87	11.79, 53.96	0.76	14.62, 99 < 0.001
	C-A	46.32	25.39, 67.26	1.26	
	C-B	13.44	(−7.48), 34.38	0.52	
Cholesterol (mg/DL)	B-A	7.39	4.87, 9.90	1.96	63.35, 99 < 0.001
	C-A	11.68	9.19, 14.18	2.60	
	C-B	4.29	1.79, 6.79	0.95	
Albumin (mg/DL)	B-A	0.31	0.16, 0.47	1.27	37.00, 99 < 0.001
	C-A	0.56	0.40, 0.71	1.93	
	C-B	0.24	0.08, 0.39	1.00	
Total protein (mg/DL)	B-A	0.58	0.27, 0.88	1.00	37.94, 99 < 0.001
	C-A	1.10	0.80, 1.40	2.30	
	C-B	0.52	0.21, 0.82	1.14	

Groups^*^: A. Control, B. playing lullaby for mother, C. playing lullaby for mother while holding a photo of her own baby

To confirm the results of the comparison between the groups, taking into account the important influencing variables in this relationship, finally the results of ANCOVA analysis for the effects of the intervention, taking into account the baseline values, showed that the intervention was effective and had the greatest effect on cholesterol levels. The results of the post hoc test also showed this significant relationship between the groups (Table 5).

**Table 5:** Evaluation of breast milk compositions after intervention with adjusted for baseline values and other variables in the study

Variable	F, df, *P* value (Effect of intervention)	*P* value (Basic value)	Adjusted R Squared	Effect size	Observed power
Volume (ml)	29.32, 2, < 0.001^a^	< 0.001	0.44	0.38	1.00
	25.36, 2, < 0.001^b^	< 0.001	0.42	0.36	1.00
Triglycerides (mg/DL)	14.73, 2, < 0.001^a^	< 0.001	0.80	0.24	0.99
	16.20, 2, < 0.001^b^	< 0.001	0.89	0.26	0.99
Cholesterol (mg/DL)	65.09, 2,< 0.001^a^	< 0.001	0.95	0.58	1.00
	67.57, 2, < 0.001^b^	< 0.001	0.96	0.59	1.00
Albumin (mg/DL)	36.46, 2, < 0.001^a^	< 0.001	0.74	0.43	1.00
	32.46, 2, < 0.001^b^	< 0.001	0.74	0.41	1.00
Total protein (mg/DL)	38.46, 2, < 0.001^a^	< 0.001	0.80	0.45	1.00
	34.67, 2, < 0.001^b^	< 0.001	0.79	0.43	1.00

^a^ Based on ANCOVA model (the included variables were: basic value of dependent variable and group type)

^b^ Based on ANCOVA model (the included variables were: basic value of dependent variable, group type, Maternal age, Birth weight, Gestational age and Maternal weight)

## Discussion

The findings of the present study showed that lullaby changed the volume and compositions of milk (triglycerides, cholesterol, albumin, total protein as well as breast milk volume) compared to routine care.

The differences and similarities of this study with previous studies are shown (Table 6).

**Table 6:** Similarities and Differences between published study and this study

Author	Title	Materials and Methods	results	similarities	differences
Ak et al. (2015 )[26]	Impact of music therapy on amount of breast milk secretion among mothers of premature newborns.	Each subject was assessed for 4 sessions on MT (Music Therapy) and 4 sessions on NMT (No Music Therapy) over 4 days. Breast milk was expressed using breast milk pump and quantity was measured for two sessions each day once at 11.00 am and other at 4.00 pm.	Music therapy group had significantly more milk volume than the control group on the fourth day and music therapy resulted in increased milk secretion in mothers of premature infants admitted to NICU.	In both studies, mothers listened to music or lullabies for 30 minutes with headphones, and then the milk was pumped through an electric pumping and measured in milliliters in a graduated bottle.	–
Keith et al. (2012 )[16]	The effect of music-based listening interventions on the volume, fat content, and caloric content of breast milk–produced by mothers of premature and critically ill infants.	The control group received standard nursing care, whereas mothers in the 3 experimental groups additionally listened to a recording of 1 of 3 music-based listening interventions while using the pump.	Mothers in the experimental groups produced significantly more milk (*P* < .0012). Mothers in these groups also produced milk with significantly higher fat content during the first 6 days of the study.	There was a significant difference between the three groups in all compositions of breast milk, which is consistent with Keith’s study, although the duration of the intervention (fourteen days in Keith’s study and six days in our study), as well as the duration of playing the music or lullaby (12 minutes in the Keith’s study and 30 minutes in our study), were different.	In our study, in addition to the volume and fat of breast milk, the total protein and albumin concentration were also measured.
Vianna et al. (2011 )[36]	Music therapy may increase breastfeeding rates among mothers of premature newborns: a randomized controlled trial.	Mothers of premature neonates weighting ≤1750 g were submitted to music therapy sessions three times a week for 60 minutes. The endpoints were breastfeeding rates at the moment of infant hospital discharge and at follow-up visits (7-15 days, 30 and 60 days after discharge).	Music therapy had a significant effect in increasing breastfeeding rates among mothers of premature newborns at the first follow-up visit, and also a positive influence (although not significant) that lasted up to 60 days after infant discharge.	Music alone can increase the volume of breast milk in mothers of premature infants admitted to NICU, which is consistent with the results of our study.	Unlike our study which was performed at the time of hospitalization of infants.
Varişoğlu et al. (2020 )[29]	The effects of listening to music on breast milk production by mothers of premature newborns in the neonatal intensive care unit: A randomized controlled study.	On the first day, all mothers were provided with training for milking with pumps. On the second through fourth days, mothers in the music group (MG) underwent two sessions of milking with music and a pump for 15 minutes at 11:00 and 16:00; the mothers in the control group underwent two sessions of milking without music. To evaluate stress levels, Spielberger’s State-Trait Anxiety Inventory was administered and salivary cortisol tests were taken on the first and final days of the study.	The state and total anxiety scores of the MG were statistically low (p < 0.05). There was no difference between the MG and control group in the amount of breast milk produced; however, the final test cortisol levels of the MG group were significantly lower compared with the pretest measurements (*p* < 0.05). Listening to music in the NICU while breastfeeding can help reduce stress levels in mothers to premature newborns and support breast milk production.	–	This difference is probably due to different intervention methods, such as the duration of the intervention, which was three days in the study of Varişoğlu et al.’s and six days in our study, as well as the duration of playing music or lullaby, which was 15 minutes in the study of Varişoğlu et al.’s and 30 minutes in our study.

Stress in mothers of hospitalized preterm infants is higher than the general population [19]. Studies have demonstrated that the first 2 weeks after birth are a critical period for the initiation and development of lactation, during which mammary epithelial cells appear to undergo programming processes that regulate long-term milk synthesis [37, 38]. Therefore, we chose the time of the baby’s hospitalization.

Maternal stress and fatigue reduce the function of oxytocin, resulting in reduced milk production and low quality [19]. Listening to music can reduce or manage stress, fatigue, and accompanying symptoms in mothers [19, 27]. Music increases oxytocin secretion which affects breast milk [16]. Oxytocin promotes prolactin release and thereby milk production [39]. Oxytocin has a positive correlation with milk volume [23]; thus, interventions that increase oxytocin secretion could lead to higher milk volume [19]. This study showed that listening and visual interventions designed to promote relaxation would prompt to reduced stress, leading to increased quantity and quality of breast milk. Although the results clearly suggest that such interventions are effective at improving quantity and quality, this study did not identify the specific causes of these changes. It is not yet clear whether the changes were due to increased oxytocin or from other factors (musical and visual experiences and oxytocin).

### Strengths and limitations

The risk of bias in our study was assessed by using the table “The Cochrane Collaboration’s tool”. The results included selection bias (random sequence generation, Criteria; using a computer random number generator-low risk, allocation concealment, Criteria; SNOSE-low risk), performance bias (Criteria; incomplete blinding, but is not likely to be influenced: measuring all outcomes in this study was objective and laboratory. Blinding was for laboratory evaluators and data analysis only -low risk), detection bias (Criteria; blinding of outcome assessment ensured -low risk), attrition bias (no missing outcome data -low risk), reporting bias (selective reporting-low risk), and other bias (Criteria; the study appears to be free of other sources of bias).

We would like to emphasize that our study has some limitations.

The first limitation was ANCOVA analysis to adjust for baseline values and other variables in this study.

The second limitation was due to the small number of target groups in this study, so it is suggested that future studies be conducted with a larger sample size. We acknowledge the limitations of conducting a single-center trial.

The third limitation, in Iranian society, the socio-economic status of individuals does not have a special ranking and is full of ambiguity and its measurement is full of errors. There is no standard measure of good or bad socioeconomic status. In this study, economic and social status was collected based on mothers’ self- report.

The fourth limitation, we recorded the birth weight of the infants from their hospital records on the first day of the study, but for the days after the intervention, their weights were not accurately recorded in the hospital records, so no weight gain was reported.

The fifth limitation was the lack of comparison between the control group and the “photo only” group. In future studies, another arm can be added, such as the ‘photo only’ group, to compare the control group with the ‘photo only’. Future studies are needed to explore the relationship to musical and visual experiences and oxytocin.

## Conclusion

Given the importance of adequate breastfeeding in the first days of an infant’s life, listening interventions such as lullabies are effective, simple, and cost-effective for mothers. We can conclude that in this small trial, there was a statistically significant association between trial arm and biochemical composition of breastmilk though further studies are needed to see if these changes result in meaningful clinical outcomes to the infant.

## Supplementary Information

**Additional file 1.**

## Data Availability

The datasets generated and analyzed during the current study are not publicly available due to an agreement with the participants on the confidentiality of the data but are available from the corresponding author on reasonable request.

## Abbreviations

NICU: Neonatal Intensive Care Unit
EBF: Exclusive Breast feeding
CI: Confidence Interval
SD: Standard Deviation
MD: Mean Difference
ANOVA: Analysis of Variance
chi2: Chi-square
SNOSE: Sequentially Numbered, Opaque Sealed Envelopes
GA: Gestational Age
LMICs: low- and Middle-Income Countries
WHO: World Health Organization
