# Supplementary material for: Effect of lullaby on volume, fat, total protein and albumin concentration of breast milk in premature infants’ mothers admitted to NICU: a randomized controlled trial
Source: Int Breastfeed J. 2022 Sep 29;17:71. doi: 10.1186/s13006-022-00511-7 (PMC9523992; doi:10.1186/s13006-022-00511-7)
Supplement: Supplementary file 1 — Additional file 1. [file 13006_2022_511_MOESM1_ESM.docx]

The secondary outcome was to determine the effect of the lullaby on cholesterol, triglyceride, total protein and albumin concentration of breast milk in mothers of preterm infants. For this purpose, the researcher collected 2 ml of breast milk from all three groups on the first and the sixth day of the study and transferred to the Biochemistry Laboratory of the Babol University of Medical Sciences to compare the biochemical indicators of breast milk in terms of fat (cholesterol and triglyceride), albumin and total protein using a Spectrophotometer with accurate measurement of 1 mg/DL performing by a fixed laboratory expert according to the instructions on the standard test kit of Pars Azmoon (made in IRAN) and based on mg/DL (Table 1).

Then the creamy top layer was removed by centrifugation at 2500 g for 12 min. Human fresh milk samples were centrifuged at 500 X g to separate milk cells and at 2500 X g to skim the milk. Skimmed milk was analyzed for albumin and total protein with the Spectrophotometric method.

After that the defatted milk was also diluted 1 in 10 with normal saline for the adaptation of our routine serum total protein and albumin methods on the Spectrophotometer.

The whole milk samples were analyzed for total protein and albumin by diagnostic test kit ( Pars azmoon ,Tehran, IRAN). The1in10 dilution was convenient for the adaptation of our routine serum triglyceride and cholesterol method on the Spectrophotometer.

The within-run precision and the between-run precision for the total protein, albumin, cholesterol and triglyceride assays were determined using whole human milk pooled from 10 donors.

Total protein was measured with Copper-based protein assays that produce azure color at 546 nm.

Results obtained using kit reagent did not show systemic differences when compared with other commercial reagents. Sensitivity was 6.6-8.8g/DL. Accuracy, The results obtained using 68 samples were shown as Regression equation: Y=1.00x - 0. 07/dl ; r=0.997.

Albumin in the presence of bromocresol green at pH=4.2, produces a color change of the indicator from yellow-green to green-blue.

Results obtained using kit reagent did not show systemic differences when compared with other commercial reagents. Sensitivity was 3. 5- 5.2 g/DL. Accuracy, The results obtained using 59 samples were shown as Regression equation: Y=1.00x - 0. 11/dl ; r=0.998

Cholesterol was determined via an enzymatic method , cholesterol ester dissociated with cholesterol esterase then cholesterol oxidized by hydrogen peroxide , then hydrogen peroxide oxidized by peroxidase enzyme 4-aminophenazone and 4-chlorophenol produced quinoneimine red color at 520 nm . Results obtained using kit reagent did not show systemic differences when compared with other commercial reagents. Sensitivity was 250 mg/DL. Accuracy, The results obtained using 57 samples were shown as Regression equation: Y=0.993x + 842/dl ; r=0.994.

Triglyceride hydrolyzed by lipase then glycerol by glycerol kinase and glycerol phosphate oxidase produced hydrogen peroxide, then hydrogen peroxide oxidized by peroxidase enzyme 4-aminophenazone and 4-cholrophenol produced quinoneimine red color.

Results obtained using kit reagent did not show systemic differences when compared with other commercial reagents. Sensitivity was 200 mg/DL. Accuracy, The results obtained using 52 samples were shown as Regression equation: Y=0.985x + 1. 893/dl ; r=0.999.
